# Influence of Fermented Broccoli Residues on Fattening Performance, Nutrient Utilization, and Meat Properties of Finishing Pigs

**DOI:** 10.3390/ani14131987

**Published:** 2024-07-05

**Authors:** Zhiwei Zhao, Jie Wu, Xiaohong Yao, Hong Sun, Yifei Wu, Hanghai Zhou, Xin Wang, Kai Guo, Bo Deng, Jiangwu Tang

**Affiliations:** 1 Institute of Plant Protection and Microbiology, Zhejiang Academy of Agricultural Sciences, Hangzhou 310021, China; zhaozhiwei990611@163.com (Z.Z.); yun6967@sina.com (X.Y.); sun_hong02@sina.com (H.S.); yifeiwu@sohu.com (Y.W.); 12034016@zju.edu.cn (H.Z.); wxxmu@163.com (X.W.); 2 Institute of Zootechnics and Veterinary Sciences, Hangzhou 310021, China; brandjerry@163.com (J.W.); guokai980219@163.com (K.G.)

**Keywords:** fermented byproducts, growth, digestibility, amino acids, fattening pigs

## Abstract

**Simple Summary:**

Residues from broccoli stems and leaves, which are generated during broccoli (*Brassica oleracea* L. *var*. *italica*) processing, are commonly discarded, contributing to environmental pollution due to their high moisture content. However, these residues are protein-rich and can be repurposed as animal feed. Fermentation further enhances their nutritional value by introducing probiotic bacteria and bioactive components. This study evaluated the influences of fermented broccoli residues (FBR) on fattening pig growth performance, nutrient utilization, and pork properties. The results demonstrated that FBR supplementation influenced meat quality, improved muscle antioxidant capacity, and reduced digestibility of finishing pigs without significantly altering amino acid composition and impacting growth and carcass traits.

**Abstract:**

The study determined the impacts of dietary fermented residues’ (FBR) inclusion on growth, nutrient utilization, carcass characteristics, and meat properties in fattening pigs. Seventy-two robust pigs were randomly assigned to two experimental groups (Duroc × Landrace × Yorkshire, thirty-six pigs each). Each group was subjected to a 52-day trial, during which they received either a corn–soybean meal-based diet or diet enhanced with a 10% addition of FBR. Consequently, adding 10% FBR caused a significant decrease in the digestive utilization of crude dietary components in fattening pigs (*p* < 0.05) but showed no significant impact on the growth performance. Additionally, FBR inclusion increased the marbling scores (*p* < 0.05) and total antioxidant functions (*p* < 0.05) of muscle tissues, indicating improved meat quality. Gender affected backfat depth, with barrows showing thicker backfat depth. In conclusion, dietary supplementation with 10% FBR in finishing pigs influenced the meat quality by improving the marbling score and antioxidant performance while reducing digestibility without compromising growth performance.

## 1. Introduction

The international farming industry is grappling with a significant challenge stemming from feed scarcity, exacerbated by an escalating demand for meat. The scarcity of feed ingredients has become more noticeable, and there has been a progressive increase in the cost of commonly used feed ingredients. This predicament has prompted exploration into cheap and alternative feed sources for livestock [1]. Broccoli, a cruciferous vegetable (*Brassica oleracea* L. var. italica), is renowned for its abundant nutritional profile, boasting essential nutrients, glucosinolates, sulforaphane, polyphenols, and minerals [2]. Notably, consuming broccoli products has been linked to human chronic disease prevention [3]. Over the past decade, broccoli cultivation has surged by 32.1%, reaching a combined production of 37.2 million tons in 2018, largely driven by China and India [4]. Nevertheless, a substantial portion of the broccoli plant, notably the leaves and stems, is commonly discarded as byproduct waste after processing, resulting in environmental pollution and resource loss due to its high perishability [5]. Recent studies have explored the application of broccoli residues as sources of bioactive compounds [6,7] and demonstrated that the extracts from leaves exhibited the highest antioxidant activities due to phenolic compounds, while stem extracts had the highest antimicrobial activity related to fatty acid derivates [8]. Another study has also found that a high protein content of 90–300 g/kg dry matter (DM) was present in broccoli residual leaves [9], but their utilization as animal feeds remains underexplored. Repurposing these broccoli residues as animal feed presents an opportunity for resource conservation and environmentally sustainable practices. Several studies have suggested that incorporating dried broccoli residues (up to 90 g/kg inclusion) into poultry diets can enhance the growth performance and improve nutrient digestibility within the digestive tract [10,11]. One study highlighted the positive impact of broccoli stem and leaf meal (up to 90 g/kg inclusion) on poultry meat quality, attributing it to its antioxidant properties [5]. Broccoli residues have shown promise as a supplementary feed in the diets of small ruminants, with the capacity to substitute up to 30% of traditional feed components without adversely affecting ruminal fermentation. This substitution is feasible, except for a slight deficiency in the supply of rumen-degradable nitrogen [12]. Moreover, fermenting plant-based materials with beneficial microbes, such as *Bacillus* and *Lactobacillus* species, can further enhance their nutritional value. In vitro findings indicate that fermentation pretreatment with *Lactiplantibacillus plantarum* is a straightforward and cost-effective technique that could lead to a boost in the nutritional content of broccoli stems and a decrease in the drying time required [13]. Animals consuming fermented broccoli residues (FBR) benefit from the inherent active compounds and the probiotic bacteria that colonize their digestive systems, providing additional health benefits [14]. Despite the fact that FBR is rich in low-molecular-weight peptides (small peptides), probiotics, and organic acids and can safeguard broilers against harmful bacterial infections when added at 5% or 10% in diets [15], the influences of FBR on other animals, particularly regarding pig growth, remain insufficiently documented. Our previous study also suggested the beneficial effect of up to 10% FBR supplementation on meat quality in growing pigs [16]. Thus, 10% might be an appropriate dosage level for dietary supplementation with FBR for its positive effects. On the other hand, it was common to use finishing pigs as experimental animals due to their high tolerance for plant protein sources and being more conducive to meat quality evaluation [17,18]. We aimed to conduct a preliminary experiment with 10% FBR inclusion and hypothesized that the dietary supplementation of 10% FBR could also influence the meat characteristics of finishing pigs without compromising their growth efficiency. Consequently, the research assessed the influence of incorporating FBR into the diet on the key metrics of finishing pigs, including growth, nutrient digestibility, and meat physiological characteristics.

## 2. Materials and Methods

### 2.1. FBR Samples

Broccoli stems and leaf residues were provided by Taizhou Tianlai Biological Technology Co., Ltd. (Taizhou, China). To produce FBR, the residues were fermented in a solid state with self-isolated strains *Bacillus pumilus*, *Enterococcus Faecium*, and *Saccharomyces cerevisiae* at a ratio of 2:1:1 at an initial addition amount of 5.0 × 10^6^ cfu/g. The fermentation substrate comprised a mixture of 60% fresh broccoli residues, 20% rice bran, and 20% corn husk [16]. Twenty-five kilograms of the substrate was kept in a plastic bag with exhaust air and sealed. Fermentation was carried out at room temperature with 50% moisture content for 21 days. The nutritional components of FBR were measured using the methods of AOAC [19], including ether extract (EE; AOAC 920.39), crude proteins (CP; AOAC 920.39), crude fibers (CF; AOAC 978.10), vitamin E (VE; AOAC 992.03), total phenolic (AOAC 2017.13). The concentrations of acid-soluble proteins (small peptides) were measured according to a previous report [20]. Briefly, approximately 2 g of FBR was homogenized with 28 mL of 5% TCA solution at 12,000 r/min for 1 min and stored at 4 °C for 30 min. The mixture was then centrifuged at 5000× *g* for 10 min to obtain the supernatant. The TCA-soluble peptide content in the supernatant was determined using a BCA protein quantification kit (Nanjing Jiancheng Biotechnology Co., Ltd., Nanjing, China). The lactic acid content was evaluated with a commercial assay kit (Nanjing Jiancheng Bio Co., Nanjing, China) according to the manufacturer’s protocol. The live microbial content was measured using traditional plate counting methods [21]. The final FBR products contained 22.9% CP, 3.37% EE, 22.19% CF, 102.32 mg/g, trichloroacetic acid-soluble protein (small peptides), 7.4 mg gallic acid/g total phenolic, 7.24 mg/kg vitamin E, 4.17% lactic acid, 4.1 × 10^8^ cfu/g *B. pumilus*, 6.7 × 10^7^ cfu/g *E. Faecium*, and 7.0 × 10^5^ cfu/g *S. cerevisiae* on an air-dry basis.

### 2.2. Animals, Diets, and Growth Performance Measurements

The research methodology was granted approval by the Committee of Laboratory Animal Welfare and Ethics of our academy (2022ZAASLA05). A total of 72 healthy fattening pigs (initial weight of 69.5 ± 1.9 kg, cross-breed of Duroc, Landrace, and Yorkshire, 36 gilts and 36 barrows) were randomly assigned to control (CON) and trial groups. Pigs designated as CON were administered a standard basal diet composed of corn–soybean meal. In contrast, the experimental group received the same basal diet with a supplementation of 10% FBR. The selection of this feeding regimen was predicated on studies that explored the influence of FBR on broilers [14] and the growth development of growing pigs [16]. Each treatment comprised six replicate pens with three males and three females per pen to minimize the influence of sex [17,20]. The basal diets were presented in a ground mash form and surpassed the nutritional requirements as required by the National Research Council [22]; they are depicted in Table 1. Pigs were accommodated in contiguous pens, each measuring 3.5 m × 3.0 m, with the ambient temperature meticulously regulated (22 °C–28 °C). The pens were also equipped with natural lighting to ensure a comfortable living environment for the animals. In the FBR group, the feed was prepared daily with a corresponding amount of FBR (in wet form), mixed according to a specific ratio (10%), and evenly distributed into the feeding trough. Throughout the 52-day trial, an ad libitum feeding regimen was implemented for all pigs, with water also being freely accessible. The pig body weights were recorded on the first and last days of the feeding trial. Average daily gain (ADG) was ascertained through the division of the total weight increase achieved throughout the experiment by the count of pigs and the total duration of the study in days. Feed intake data were recorded on a per-pen basis daily for estimating the daily feed intake (ADFI). The proportion of ADG to ADFI was calculated to present the gain-to-feed ratio (G/F).

### 2.3. Nutrients’ Digestibility

The nutrients’ digestibility was ascertained utilizing the acid-insoluble ash (AIA) technique [23]. Briefly, fecal samples were collected daily in replicate from two pigs via rectal massage for three consecutive days (from day 49 to day 51), pooled, and treated with 10% sulfuric acid at a ratio of 10 mL per 100 g of feces to mitigate nitrogen loss through evaporation. Subsequently, the fecal samples were dried, followed by pulverization to achieve particle sizes suitable for sieving, and subjected to nutrient analysis. The concentrations of DM, CP, CF, as well as the calcium and total phosphorus in both fecal and feed samples were measured in accordance with the protocols of AOAC [19]. Subsequently, the apparent total tract digestibility (ATTD) was deduced using a predefined formula, as referenced in the literature [24]:

(1) $$ATTD\left(\%\right)=100-(100\times \frac{\%{AIA}_{diet}}{\%{AIA}_{fecal}}\times \frac{\%N{utrients}_{fecal}}{\%{Nutrients}_{diet}})$$

### 2.4. Carcass Characteristics

Pigs were fasted for 24 h after the completion of the dietary trial, and one finishing pig per replicate (pen) with similar body weight was chosen for carcass characteristics measurement (three barrows and three gilts/per treatment). In brief, pigs were electrically stunned, exsanguinated, and eviscerated at a nearby slaughter. Carcass weight measurement was taken. The dressing efficiency was ascertained by the ratio of carcass weight to the weight of the pig at the time of slaughter. The area of the loin muscle and average backfat thickness were determined as previously described [25]. Longissimus thoracis (LT) were meticulously harvested from the left side of the carcass after tissue and subcutaneous fat cleaning for color, pH, marbling score, drip loss, shear force, and antioxidant parameter analyses. Another portion of the LT (approximately 500 g) was nitrogen-frozen. The samples were kept in a cryogenic freezer maintained at −80 °C until the detection of chemical profile and fiber-type-related gene expression.

### 2.5. The Quality of Meat and its Amino Acid Profile

#### 2.5.1. Meat Quality

Post-mortem muscle pH was measured at two distinct time intervals—45 min (designated as pH_45min_) and 24 h (designated as pH_24h_)—utilizing a portable pH meter manufactured by Fisher Scientific (Pittsburgh, PA, USA). Concurrently, meat color attributes, including redness (a*), lightness (L*), and yellowness (b*), were assessed by utilizing a Minolta CR-410 Chroma Meter at precisely 45 min after slaughter. The marbling score was measured according to the scoring chart provided by the NPPC [26]. The drip loss evaluation was conducted by securing muscle tissue samples within a plastic bag and subjecting them to a 48 h refrigeration period at a temperature of 4 °C within 2 h after slaughter [27]. The muscle sample was reweighed after absorbing surface water using filter papers. The tenderness of the meat (shear force) was evaluated using the C-LM3 shear force device from Tenovo, Beijing, China, parallel to the muscle fibers’ direction on muscle sample cuts with a size of 1 × 1 × 2 cm in length, width, and height. Intramuscular fat was measured via ether extraction. Each measurement was carried out in triplicate.

#### 2.5.2. Antioxidant Capability

A 1 g muscle sample was homogenized in a 10 mL solution of sterilized 0.9% saline. The homogenate was then centrifuged at 3000 r/min for a duration of 10 min, with the process maintained at 4 °C. The antioxidant properties of the resulting supernatant were performed. The levels of malondialdehyde (MDA), as well as the activities of catalase (CAT) and superoxide dismutase (SOD), were determined from the supernatant. Additionally, the overall antioxidant capacity (T-AOC) of the muscle tissue was determined. All assays were conducted utilizing assay kits procured from the Nanjing Jiancheng Bioengineering Institute, Nanjing, China, following the protocols specified by the manufacturer.

#### 2.5.3. Amino Acid Profiles

Amino acid composition was measured as previously reported [26]. Briefly, approximately 100 mg of dried muscle was placed in glass bottles, kept at 110 °C, and hydrolyzed with 6 mol/L HCl for a duration of 24 h. The prepared combination was carefully transferred into a glass flask (50 mL). Subsequently, it was purified by filtration through a membrane filter (0.22 μm) to ensure sterility and remove particulates. The resulting filtrate, an aliquot of 1 mL, was concentrated by evaporation in a 60 °C water bath. After evaporation, the concentrate was re-dissolved in a 0.02 mol/L hydrochloric acid solution and then refrigerated at 4 °C to preserve its integrity for subsequent analysis. The amino acid concentration of the solution was then delineated using the facilities of a HITACHI automated analyzer (L-8900 Hitachi, Tokyo, Japan). The analysis was conducted by strictly following the operational guidelines provided by the producer. The conditions were set as follows: the chromatographic column was cation exchange resin (4.6 mm × 60 mm) with a column oven temperature of 57 °C; the injection volume was 20 μL, and detection wavelength was 570 nm/440 nm; the mobile phase was citric acid-sodium citrate with a flow rate of 0.25 mL/min; the chromogenic agent was ninhydrin solution with a flow rate of 0.125 mL/min, and the temperature of derivatization was 135 °C.

### 2.6. Real-Time PCR Quantification

The extraction of total RNA, synthesis of complementary DNA (cDNA), and quantification through real-time PCR for muscle samples were executed according to established methodologies [25,26]. In brief, the whole RNA was extracted from the tissue samples using Takara TRIzol reagent (Dalian, China). The concentration and purity of the extraction were determined by a NanoDrop 2000 spectrophotometer (ThermoFisher Scientific, Waltham, MA, USA), with RNA integrity confirmed via the 260/280 nm absorbance ratio. The values obtained were within acceptable limits, specifically ranging from 1.8 to 2.0. Subsequently, cDNA was synthesized from the purified RNA utilizing a First Strand cDNA Synthesis Kit (6110A, Takara, Tokyo, Japan), following the protocol provided by the manufacturer. Real-time PCR analysis was conducted on a StepOnePlus station (Applied Biosystems, CA, USA). A SYBR Ex Taq II kit (RR820A, Takara, Tokyo, Japan) was employed for fluorescence detection. Primers specific for myosin heavy chain (*MyHC*) *I*, *IIa*, *IIx*, and *IIb*, were as reported in previous studies [25]. Gene quantifications were conducted using the comparative CT method (2^−ΔΔCT^), with the pig *GAPDH* gene (GenBank No. NM_001206359.1) serving as the endogenous control for normalization. The PCR amplification protocol involved a start step at 95 °C for 60 s for denaturation. For amplification, 40 cycles were conducted, with each cycle including a denaturation step at 95 °C for 15 s and an annealing/extension step at 60 °C for 30 s. Each detection was conducted in triplicate.

### 2.7. Statistical Analysis

The results are presented as mean values, each accompanied by its respective standard error. Data analysis was conducted utilizing the SPSS 13.0 statistical software (version 13.0, Chicago, IL, USA). For growth and nutrient digestibility parameters, we employed Student’s *t*-test for statistical comparison, and the pen was designated as the experimental unit. For the meat quality parameter, a 2 × 2 factorial arrangement consisting of treatment and gender was used for analysis. Individual pigs were considered the statistical unit for meat quality parameter analyses. *p* < 0.05 was determined as the statistical significance level and 0.05 < *p* < 0.10 as a significant trend.

## 3. Results

### 3.1. Fattening Performance

The influence of FBR supplementation on the growth efficiency of fattening pigs is presented in Table 2. No significant disparities were found in growth performance (final body weight, ADG, ADFI, and G/F) between the CON and FBR groups throughout the experimental periods (*p* > 0.05).

### 3.2. Nutrient Utilization

The influence of dietary supplementation of FBR on ATTD of DM, CP, CF, calcium, and total phosphorus in fatteners is presented in Table 3. The digestibility of DM, CP, and CF was significantly lower in pigs receiving 10% FBR compared to pigs in the CON group (*p* < 0.05). However, no significant differences in the apparent digestibility of calcium and total phosphorus were found between the two groups (*p* > 0.05).

### 3.3. Carcass Performances and Meat Properties

As delineated in Table 4, supplementation of FBR markedly increased the marbling scores (*p* < 0.05) and showed a tendency to decrease the drip loss (*p* = 0.074) of LT compared to the CON group. In congruence, the T-AOC of LT exhibited a marked elevation in the FBR group (*p* < 0.05). The gender had a significant impact on carcass characteristics. Gilts showed a significantly decreased backfat depth (*p* < 0.05) and a trend to lower muscle lightness (*p* = 0.09). Additionally, gender and supplementation of FBR showed a significant interaction with backfat depth and shear force (*p* < 0.05).

### 3.4. Amino Acids

The amino acid composition of the longissimus muscle is summarized in Table 5. FBR supplementation or gender did not significantly influence the amino acid composition in the longissimus dorsi muscle, including the concentrations of total flavor amino acids, essential amino acids, and total amino acids (*p* > 0.05).

### 3.5. Muscle Fiber Type

Compared to the CON group, dietary FBR supplementation had no significant influence on MyHC I, MyHC IIa, MyHC IIb, and MyHC IIx expression (*p* > 0.05) in the longissimus muscle of pigs fed diets supplemented with FBR (Table 6). Gender and supplementation of FBR showed a tendency interaction in MyHC IIx expression (*p* = 0.093).

## 4. Discussion

Given our present insights, this is the inaugural investigation into the impact of FBR on finishing pigs. The absence of notable variances in final body weights, ADFI, ADG, and G/F between the CON and FBR groups suggests that incorporation of 10% FBR into the diet exhibited no adverse influence on the fattening performance of fattening pigs. These results may somewhat contrast with a previous study [15] showing that supplementing 25, 50, and 75 g/kg FBR significantly increased the ADG of broilers. Additionally, our previous research [16] indicated that supplementing 5% FBR significantly increased the ADFI of growing pigs. These discrepancies can be attributed to the distinct FBR levels and animals involved. Our results align with a previous study [14] showing that supplementation of 10% FBR did not notably impact the ADG and feed utilization of broilers. The potential reason could be that dietary fiber-rich materials, such as broccoli residues, stimulate the expansion of visceral organs and the production of digestive juices, consequently allocating more energy toward maintenance rather than catabolic metabolism, especially for pigs in the finishing phase [28]. Thus, our results indicate that FBR may be effectively integrated into the dietary regimen of finishing pigs at concentrations up to 10% without detrimental effects. Given the scarcity of research assessing the impact of fermented broccoli products on livestock, it is not feasible to draw any additional comparisons.

The digestibility of nutrients serves as a crucial indicator linked to the growth performance of animals. In our study, FBR supplementation in finishing pig diets significantly decreased the nutrient utilization of CP, DM, and CF, consistent with a study showing a drop in the digestive efficiency of pigs during the growth-to-finishing phase when fed high-fiber diets [27]. Similarly, Mustafa and Baurhoo [10] noted a markedly decreased apparent ileal digestibility of CP and DM in 35-day-old birds when their diet included 10% broccoli residues. Our previous research in growing pigs also indicated a trend (0.05 < *p* < 0.1) toward reduced nutrient digestibility when supplementing 5% and 10% FBR [16]. Moreover, several investigations have shown that incorporating fermented feeds, such as fermented soybean meal and wheat bran, into the diets of growing or finishing pigs leads to better nutrient absorption [20,23,25], as fermentation can degrade complex macro-molecules into small substances, promoting feed digestion [29]. On the other hand, increasing dietary fiber could decrease nutrient digestibility by reducing nutrient digestion time in the gastrointestinal tract [30,31]. Thus, the inconsistencies observed across these studies can likely be ascribed to the elevated levels of dietary fiber present in the experimental diets, which impedes nutrient breakdown and uptake during digestion and counteracts the positive impact of probiotics on intestinal digestive enzymes [32]. Furthermore, diet composition, the anti-nutritional factors present in FBR, variations in bacterial strains, and experiment duration may all contribute to the variability observed across different studies. Interestingly, although significant inhibition in digestibility of CP, DM, and CF was observed in pigs fed with FBR-supplemented diets, the pig growth was not adversely impacted under the conditions of this study. Previous studies found that *Bacillus* spp. and *Lactobacillus* sp. could increase the activities of digestive enzymes, promote nutrient transporter expression, and improve intestinal morphology [33]. Thus, the presence of live micro-organisms in the FBR might contribute, in part, to the compensatory effects on decreased digestibility in finishing pigs. Moreover, acid-soluble proteins (small peptides) are largely present in FBR, which facilitates the uptake of nutrients in the digestive system of animals, thereby offsetting the adverse effects of high crude fiber content in diets [17]. Further research is needed to explore whether FBR influences the intestine structure and nutrient absorption to better understand this phenomenon. 

Slaughter performance metrics serve as vital criteria for evaluating the performance of meat carcasses. We reported that the dietary inclusion of FBR had no adverse impact on the carcass performance of finishing pigs. The finding aligns well with our previous study [16], which demonstrated no adverse influence on carcass characteristics of pigs in the growing stage fed FBR at up to 10% inclusion levels. The marbling score, a key indicator of fat deposition in pork muscles, is closely correlated with meat eating quality, including flavor and tenderness [34]. Our finding demonstrated a significant elevation in marbling scores in the FBR-supplemented group, indicating improved meat quality. This observation is consistent with the data presented by Hao et al. [20] and Liu et al. [26], who reported increased marbling scores or intramuscular fat content in pigs in the finishing phase fed diets supplemented with fermented mixed feed. The abundance of probiotic strains present in FBR, such as *Bacillus* sp. and *Lactobacillus* sp., may contribute to these beneficial effects on meat quality [35]. The optimization of gut microbiota by probiotics after dietary FBR inclusion may lead to enhanced nutrient absorption and improved efficiency of converting feed into body mass, which in turn could elevate the overall metabolism of nutrients, resulting in an increased marbling score [28]. Additionally, some *Lactobacillus* strains were found to produce vitamin E, which contributes to the increase in the marbling score [36]. The marbling score is an indicator of both the quantity and the distribution pattern of fat within the muscle tissue, correlating positively with the content of intramuscular fat [18]. Interestingly, the present study reported that the marbling score of the longissimus muscle exhibited a notable increase, without corresponding to any significant alteration in the intramuscular fat content, indicating that FBR mainly affected the fat distribution pattern in muscle. Similar discrepancies between marbling and intramuscular fat were observed in other studies [17,18], and their correlation needs further investigation. Our results also showed that gender significantly changed the backfat depth. Barrows had a thicker backfat depth than gilts, indicating a lower leanness in barrows. Similar results were also shown in previous studies [37,38], suggesting that barrows use more energy on fat deposits than muscle deposits.

Increased muscle antioxidant ability has been observed in FBR-fed finishing pigs [39]. Our study also revealed significantly increased T-AOC contents in the muscles of FBR-fed pigs, suggesting a potential protective effect of FBR against muscle oxidative damage. Similar studies have demonstrated that supplementing FBR improved SOD activity in longissimus dorsi muscle in pigs [20] and SOD and CAT activities and T-AOC in pectoralis major muscle and gastrocnemius muscle in free-range broilers [14]. A previous study demonstrated that dietary inclusion of broccoli stem and leaf meal at a concentration of 4%, 8%, or 12% significantly improved muscle T-AOC of Ross 308 male broilers [5]. Due to limited research on the impact of FBR on the antioxidant status of meat, further comparisons are challenging. The abundance of probiotics in FBR may partly explain the improvement in antioxidant capability in pigs [40]. The genus *Lactobacillus* exhibits dose-responsive free radical scavenging capabilities, and its supplementation has been shown to enhance the antioxidant status of finishing pigs [41]. Nevertheless, small peptides in FBR also contribute to the improvement in muscle antioxidant ability, as a previous study reported that a dipeptide (carnosine) could enhance the oxidative activity of pigs’ muscle [42]. Additionally, bioactive molecules produced during the solid-state fermentation of broccoli residues, such as flavonoid and phenolic compounds, have been reported to increase sharply during lactic acid bacteria fermentation [2,6]. Given the close relationship between meat shelf life and antioxidant capacity, FBR appears to function as a functional feed to elevate the quality of meat by improving the antioxidant status of the longissimus muscle. Furthermore, dietary inclusion of FBR did not yield any significant effect on meat color, which conflicts with our previous findings regarding the influence of FBR on growing pigs [16]. This result somewhat agrees with a previous study indicating that dietary inclusion of fermented soybean meal does not affect meat color parameters [25]. Previous studies have shown that the impact of fermented feed on the meat properties of pigs in the growing–finishing stage follows a concentration-dependent pattern [27,43]. The FBR supplementation level in our current study may have been too low to significantly affect meat color, as previous studies demonstrated that there was a positive correlation between the effect on meat color and the dietary concentrations of fermented feed [44]. Further research employing higher levels (>10%) of FBR supplementation is necessary to better understand the influence of FBR supplementation on meat color in finishing pigs.

The amino acid composition of muscle tissue is intricately linked to meat taste and nutritional benefits [45]. Recent studies have revealed that dietary supplementation with fermented feed effectively increases amino acid concentrations, thereby improving meat quality [46,47]. Liu et al. [26] demonstrated that pigs fed fermented mixed feed had increased levels of methionine, lysine, threonine, alanine, aspartate, glutamate, arginine, and total essential amino acids. Tang et al. [48] demonstrated that finishing pigs fed fermented feed had higher lysine and glutamate contents than those fed a basal diet. However, in our study, FBR supplementation did not significantly affect longissimus dorsi muscle’s amino acids. The result somewhat aligns with previous research [49], which reported no significant benefits of fermented feed on the essential amino acid profile of muscle. These discrepancies may be attributed to differences in experimental variables, such as breed and age of the experimental animals, fermented substrates, or fermentation strains used. Given that the precise regulatory mechanism governing amino acid content is not yet fully understood, our results suggest that dietary supplementation of FBR has no adverse influence on the amino acid profiles of finishing pigs.

Muscle fibers, serving as the fundamental units of muscle tissue, are categorized into four distinct categories: Type I, distinguished by their slow-twitch characteristics; Type IIa, exhibiting fast-twitch properties; Type IIx, also known for its fast-twitch nature; and Type IIb, which shares the fast-twitch attribute. This categorization was based on the unique expression patterns of MyHC isoforms present in each fiber type [50]. The assortment and relative distribution of muscle fiber types are intricately linked to the attributes of muscle color, texture, and flavor, thereby exerting a pivotal effect on the overall quality of meat [51]. A prior study revealed that the inclusion of fermented feed in the diet substantially elevated the mRNA expression levels of *MyHC I* and *MyHC IIa*, indicating a potential shift from the fast-twitch to the slow-twitch muscle fiber phenotype [18]. As is widely recognized, the reduction in the proportion of glycolytic muscle fibers and a concurrent rise in oxidative fibers in muscle tend to result in meat with a more intense red color, as oxidative fibers contain more pigment protein myoglobin than glycolytic fibers [50]. However, our result showed that FBR supplementation had no significant effects on fiber gene expression level, suggesting no such benefits from FBR. Several signal transduction pathways have been implicated throughout the metamorphosis of muscular fiber classifications, including calcineurin and nuclear factor of activated T-cell c1 pathway [52], mechanistic target of rapamycin kinase pathway [53], and AMP-activated protein kinase pathway [54]. We speculated that this discrepancy may be partly attributed to these influence factors. However, the specific process still warrants further research.

## 5. Conclusions

Taken together, dietary FBR supplementation (10%) in fattening pigs had no impact on the growth and carcass performance but affected the quality of meat by improving the marbling score and enhancing the antioxidant performance. Adding FBR also significantly reduced the apparent digestibility of finishing pigs. The results indicate that FBR could be applied as a feed ingredient in finishing pig diets. The experiment lays a groundwork for FBR application in finishing pigs, although further investigation into broader dose levels is warranted to maximize its effects.

## Figures and Tables

**Table 1:** Nutritional profile based on ingredient composition (air-dry percentages).

Ingredients	CON ^1^	FBR ^1^
Corn (7.8% CP)	75.00	72.45
Soybean meal (43% CP)	14.62	13.12
Wheat bran	6.00	0.00
FBR	0.00	10.00
Lys Hydrochloride (98.5%)	0.20	0.25
DL-Met (99%)	0.10	0.10
L-Thr (99%)	0.08	0.08
Vitamin–mineral premix ^2^	4.00	4.00
Total	100	100
Nutrient composition ^3^		
Dry matter (%)	87.21	83.60
Digestible energy (MJ/kg)	13.09	13.02
Crude protein	13.91	13.94
Crude fiber	2.85	4.40
Lysine	0.78	0.78
Calcium	0.60	0.62
Total phosphorus	0.48	0.48

^1^ CON: control; FBR: fermented broccoli residues. ^2^ Provided the following (per kilogram of complete diet): iron, 60 mg; copper, 5 mg; zinc, 50 mg; manganese, 20 mg; selenium, 0.3 mg; iodine, 0.5 mg; vitamin A, 4500 IU; vitamin D3, 600 IU; vitamin E, 38.5 mg; vitamin K3, 1.35 mg; vitamin B1, 2.5 mg; vitamin B2, 6.5 mg; vitamin B6, 3.0 mg; vitamin B12, 0.025 mg; niacin, 25 mg; folic acid, 15 mg; biotin, 0.75 mg. ^3^ Digestible energy in nutritional levels is calculated, while other indices are analyzed values.

**Table 2:** Growth performance of finishing pigs in CON and FBR groups.

Items	CON ^1^	FBR ^1^	*p*-Value
Initial body weight (kg)	69.55 ± 2.90	69.42 ± 2.85	0.975
Final body weight (kg)	111.50 ± 3.38	107.69 ± 2.69	0.398
Average daily gain (g/d)	757.3 ± 17.7	716.5 ± 27.6	0.242
Feed intake (g/d)	2486.1 ± 60.8	2522.1 ± 96.0	0.758
Gain-to-feed ratio	0.30 ± 0.01	0.29 ± 0.01	0.135

^1^ CON: control; FBR: fermented broccoli residues. The presented data are articulated as mean values, each accompanied by its respective standard error (n = 6).

**Table 3:** Nutrient ATTD of finishing pigs in CON and FBR groups.

Items (%)	CON ^1^	FBR ^1^	*p*-Value
Dry matter	87.07 ± 0.43 ^a^	82.38 ± 0.32 ^b^	<0.001
Crude protein	79.79 ± 0.76 ^a^	74.47 ± 0.38 ^b^	<0.001
Crude fiber	65.42 ± 1.36 ^a^	60.76 ± 1.09 ^b^	0.023
Calcium	55.48 ± 1.44	49.96 ± 3.09	0.136
Total phosphorus	62.45 ± 0.96	65.05 ± 2.01	0.268

^1^ CON: control; FBR: fermented broccoli residues. ^a,b^ Mean values in the same row that are annotated with distinct superscripts are significantly different (*p* < 0.05). The data are articulated as mean values, each accompanied by its respective standard error (n = 6).

**Table 4:** Carcass performance and meat quality of LT of pigs in CON and FBR groups.

Items	Treatment		Gender		*p*-Value		
	CON ^1^	FBR ^1^	Barrows	Gilts	Treatment	Gender	Treatment × Gender
Carcass characteristics							
Carcass weight (kg)	97.75 ± 1.84	97.61 ± 1.36	97.98 ± 1.54	97.38 ± 1.40	0.959	0.818	0.693
Dressing percentage (%)	76.45 ± 0.87	76.57 ± 0.24	77.15 ± 0.55	75.86 ± 0.49	0.878	0.123	0.101
Loin muscle area (cm^2^)	49.22 ± 2.16	49.20 ± 2.53	47.31 ± 1.65	51.11 ± 2.31	0.996	0.290	0.472
Backfat depth (cm)	3.37 ± 0.03	3.47 ± 0.11	3.53 ± 0.07 ^a^	3.30 ± 0.04 ^b^	0.195	0.011	0.046
pH_45 min_	6.01 ± 0.04	6.11 ± 0.10	6.00 ± 0.05	6.12 ± 0.08	0.360	0.237	0.226
pH_24 h_	5.74 ± 0.04	5.71 ± 0.07	5.70 ± 0.05	5.75 ± 0.06	0.754	0.621	0.754
Color							
Lightness (L*)	56.60 ± 1.77	56.05 ± 1.50	58.31 ± 1.38	54.34 ± 1.13	0.796	0.090	0.363
Redness (a*)	17.39 ± 0.19	17.14 ± 0.21	17.43 ± 0.07	17.10 ± 0.24	0.384	0.273	0.332
Yellowness (b*)	7.72 ± 0.40	7.05 ± 1.11	7.66 ± 0.42	7.11 ± 0.37	0.336	0.421	0.879
Drip loss (%)	4.49 ± 0.16	3.38 ± 0.50	3.63 ± 0.33	4.23 ± 0.44	0.074	0.298	0.782
Marbling scores	1.71 ± 0.12 ^b^	2.54 ± 0.20 ^a^	2.04 ± 0.25	2.21 ± 0.20	0.010	0.524	0.747
Intramuscular fat (%)	3.07 ± 0.13	3.33 ± 0.16	3.10 ± 0.14	3.30 ± 0.13	0.261	0.377	0.639
Shear force (N)	40.40 ± 1.02	38.74 ± 0.94	39.24 ± 1.08	39.91 ± 0.79	0.176	0.567	0.029
Antioxidant parameters ^2^							
MDA (nmoL/mg prot)	0.40 ± 0.14	0.24 ± 0.11	0.18 ± 0.08	0.33 ± 0.12	0.396	0.850	0.458
CAT (U/mg prot)	0.15 ± 0.03	0.12 ± 0.01	0.15 ± 0.03	0.13 ± 0.01	0.586	0.855	0.471
T-AOC (U/mg prot)	0.16 ± 0.03 ^b^	0.27 ± 0.02 ^a^	0.19 ± 0.04	0.23 ± 0.03	0.040	0.835	0.576
SOD (U/mg prot)	118.03 ± 12.26	113.86 ± 7.01	119.35 ± 8.28	112.54 ± 9.76	0.663	0.516	0.672

^1^ CON: control; FBR: fermented broccoli residues. ^2^ MDA: malonaldehyde; T-AOC: total antioxidant capacity; CAT: catalase; SOD: superoxide dismutase. ^a,b^ Mean values in the same row that are annotated with distinct superscripts are significantly different (*p* < 0.05). The data are articulated as mean values, each accompanied by its respective standard error (n = 6).

**Table 5:** Amino acid composition of LT of pigs in CON and FBR groups (as-fresh basis, g/kg).

Items	Treatment		Gender		*p*-Value		
	CON ^1^	FBR ^1^	Barrows	Gilts	Treatment	Gender	Treatment × Gender
Essential amino acids							
Valine	11.19 ± 0.15	11.05 ± 0.53	11.40 ± 0.45	10.83 ± 0.27	0.824	0.351	0.705
Threonine	9.05 ± 0.25	8.94 ± 0.35	8.87 ± 0.41	8.65 ± 0.22	0.233	0.637	0.533
Isoleucine	8.52 ± 0.20	8.28 ± 0.43	8.65 ± 0.39	8.15 ± 0.24	0.648	0.354	0.924
Methionine	5.07 ± 0.45	3.98 ± 0.36	4.55 ± 0.48	4.49 ± 0.47	0.107	0.922	0.270
Histidine	9.57 ± 0.43	9.43 ± 0.46	9.69 ± 0.48	9.31 ± 0.39	0.820	0.556	0.198
Phenylalanine	6.56 ± 0.10	6.29 ± 0.42	6.61 ± 0.38	6.23 ± 0.19	0.573	0.424	0.630
Lysine	23.73 ± 1.43	22.93 ± 1.87	24.07 ± 1.63	22.59 ± 1.64	0.762	0.574	0.676
Leucine	14.41 ± 0.33	13.57 ± 0.97	14.12 ± 1.00	13.86 ± 0.36	0.473	0.824	0.600
Non-essential amino acids							
Alanine	12.84 ± 0.27	12.16 ± 0.57	12.72 ± 0.48	12.27 ± 0.43	0.351	0.526	0.837
Aspartate	16.05 ± 0.70	15.33 ± 1.95	16.39 ± 1.87	14.98 ± 0.81	0.757	0.549	0.937
Glutamate	22.94 ± 0.77	21.21 ± 1.06	22.52 ± 1.06	21.62 ± 0.91	0.254	0.542	0.624
Arginine	9.51 ± 0.75	8.09 ± 0.71	8.97 ± 0.65	8.62 ± 0.91	0.237	0.764	0.449
Glycine	6.75 ± 0.16	6.09 ± 0.36	6.38 ± 0.38	6.47 ± 0.22	0.158	0.836	0.727
Serine	5.88 ± 0.10	5.42 ± 0.28	5.76 ± 0.22	5.53 ± 0.24	0.191	0.499	0.885
Tyrosine	3.98 ± 0.48	4.12 ± 0.40	4.20 ± 0.39	3.90 ± 0.48	0.822	0.646	0.217
Proline	7.14 ± 0.12	6.97 ± 0.34	7.19 ± 0.31	6.91 ± 0.16	0.658	0.492	0.617
Cysteine	0.75 ± 0.07	0.65 ± 0.04	0.74 ± 0.06	0.66 ± 0.05	0.199	0.344	0.306
Total essential amino acids	78.52 ± 2.45	74.56 ± 3.67	78.28 ± 3.22	74.80 ± 3.09	0.432	0.488	0.998
Flavor amino acids ^2^	68.09 ± 5.82	62.88 ± 9.08	66.99 ± 3.41	63.97 ± 3.09	0.309	0.547	0.946
Total amino acids	173.94 ± 5.15	164.02 ± 8.05	84.89 ± 4.11	80.99 ± 3.36	0.328	0.504	0.940

^1^ CON: control; FBR: fermented broccoli residues. ^2^ Flavor amino acids: glycine, alanine, aspartate, glutamate, and arginine. The data are articulated as mean values, each accompanied by its respective standard error (n = 6).

**Table 6:** The expression levels of genes associated with muscle fiber types of finishing pigs in CON and FBR groups.

Items	Treatment		Gender		*p*-Value		
	CON ^1^	FBR ^1^	Barrows	Gilts	Treatment	Gender	Treatment × Gender
MyHC IIx	1.20 ± 0.31	1.39 ± 0.18	1.08 ± 0.24	1.50 ± 0.20	0.549	0.218	0.093
MyHC I	1.11 ± 0.21	1.37 ± 0.25	1.15 ± 0.20	1.32 ± 0.22	0.497	0.646	0.692
MyHC IIa	1.10 ± 0.20	1.54 ± 0.26	1.08 ± 0.22	1.55 ± 0.20	0.214	0.179	0.842
MyHC IIb	1.58 ± 0.77	1.47 ± 0.16	1.23 ± 0.23	1.82 ± 0.66	0.891	0.495	0.449

^1^ CON: control; FBR: fermented broccoli residues. The data are articulated as mean values, each accompanied by its respective standard error (n = 6).

## Data Availability

Upon a legitimate and reasonable request, the data can be made accessible through the corresponding authors.
